# Minimally invasive medial maxillectomy and the position of nasolacrimal duct: the CT study

**DOI:** 10.1007/s00405-016-4376-8

**Published:** 2016-11-14

**Authors:** Andrzej Sieskiewicz, Krzysztof Buczko, Jacek Janica, Adam Lukasiewicz, Urszula Lebkowska, Bartosz Piszczatowski, Ewa Olszewska

**Affiliations:** 1 Department of Otolaryngology, Medical University of Bialystok, Sklodowskiej-Curie 24A, 15-276 Bialystok, Poland; 2 Department of Radiology, Medical University of Bialystok, Bialystok, Poland

**Keywords:** Maxillary sinus, Nasolacrimal duct, Medial maxilectomy, Lacrimal recess

## Abstract

Several minimally invasive modifications of endoscopic medial maxillectomy have been proposed recently, with the least traumatic techniques utilizing the lacrimal recess as a route to enter the sinus. The aim of the study was to analyze the anatomy of medial maxillary wall in the region of nasolacrimal canal and, thus, to determine the capability of performing minimally invasive approach to the maxillary sinus leading through the lacrimal recess. The course of nasolacrimal canal and the distance between the anterior maxillary wall and the nasolacrimal canal (the width of lacrimal recess) were evaluated in 125 randomly selected computed tomography (CT) head examinations. The proportion of cases with unfavorable anatomical conditions (lacrimal recess too narrow to accept a 4 mm optic) to perform minimally invasive middle maxillectomy was assessed. The width of lacrimal recess, measured at the level of the inferior turbinate attachment, varied between 0 and 15.2 mm and was related to slanted course of nasolacrimal canal. The more perpendicular the axis of the canal to the nasal flor, the narrower the lacrimal recess. In about 16% of cases, lacrimal recess width was less than 4 mm and in 14.4% it was missing. The endoscopic approach to maxillary sinus leading through lacrimal recess is possible in about 70% of patients. In the remaining group of patients when the lacrimal recess is too narrow, this type of approach may be difficult to perform without damaging the piriform aperture rim or bony framework of nasolacrimal duct, or it may be impracticable when lacrimal recess is missing.

## Introduction

The transnasal endoscopic middle antrostomy is currently the most favored and most commonly performed procedure for surgical treatment of inflammatory lesions of the maxillary sinus. Although this type of approach to the maxillary sinus allows for overall good visualization of the operated sinus, the normal sized middle antrostomy does not allow for endoscopic inspection of the entire sinus even using angled optics. Moreover, without extensive excision of the lateral nasal wall inferiorly and anteriorly, including the nasolacrimal duct, over half of the maxillary sinus may remain out of reach with commercially available instruments [1–3].

To improve the access to the maxillary sinus and to avoid unnecessary trauma related to maximal enlargement of natural ostium or entering the sinus through canine fossa, various minimally invasive modifications of medial maxillectomy with preservation of nasolacrimal duct have been proposed recently [4–6]. In these procedures, the maxillary sinus is explored through the opening created in its medial wall, anteriorly to the nasolacrimal duct, i.e., through lacrimal recess. It has been proved that this approach considerably improves visualization of the antero-inferior part of the maxillary sinus and it is most difficult to reach dental and lacrimal recesses.

Although the advantages of minimally invasive endoscopic medial maxillectomy have been discussed extensively by many authors, to the best of our knowledge, no one has analyzed the variability of anatomical conditions determining feasibility of this type of approach.

The location of the nasolacrimal duct in relation to anterior maxillary wall determines the width of the lacrimal recess. It varies between individuals making surgery technically easy in patients with wide lacrimal recess or demanding or impossible if lacrimal recess is narrow or missing.

The purpose of this study was to analyze the course of nasolacrimal duct and assess the distance between nasolacrimal duct and anterior maxillary wall based on computed tomography studies and, thus, to determine the capability of performing minimally invasive approach to the maxillary sinus leading through lacrimal recess.

## Materials and methods

The studied group consisted of 125 patients, 250 measurements (left and right side in 52 M and 73 F, aged 18–76 years, on average 44 years) who were randomly selected from our CT database. Signs of congenital maxillofacial malformations or history of trauma of this region as well as signs of chronic inflammatory disease of maxillary sinus were considered as exclusion criteria. The mean age of males was 39 years (range 18–76 years) and that of females was 48 years (range 19–75 years).

Aquilion CX multidetector row CT scanner (Toshiba, Medical Systems, Tokyo, Japan) equipped with 64 × 0.5 mm detector rows was used for all examinations. The data acquisition was performed according to an established protocol of the helical mode. The parameters included a 120 kV tube voltage, 75 effective mAs, 0.5 s rotation time, section thickness of 0.5 mm, and a field of view (FOV) of 240 mm^2^. For all examinations, unenhanced volume CT was routinely performed. The patient was imaged in the supine position for axial sections—parallel to the hard palate. The scanning length covered cranium from frontal sinus to mandible. From the raw data, coronal reformations were reconstructed (perpendicular to the hard palate) on the console or using dedicated workstation software (Vitrea fx version 5.0; Vital images, Inc., Minnetonka, MN, USA).

**Fig. 1:**
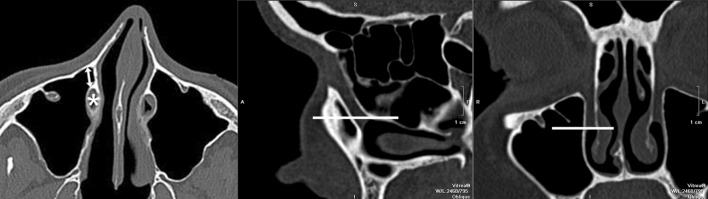
The level at which the measurements were taken—axial plane (*left*), sagittal plane (*middle*) and coronal plane (*right*), nasolacrimal duct (*asterix*), the width of lacrimal recess (*arrow*)

**Fig. 2:**
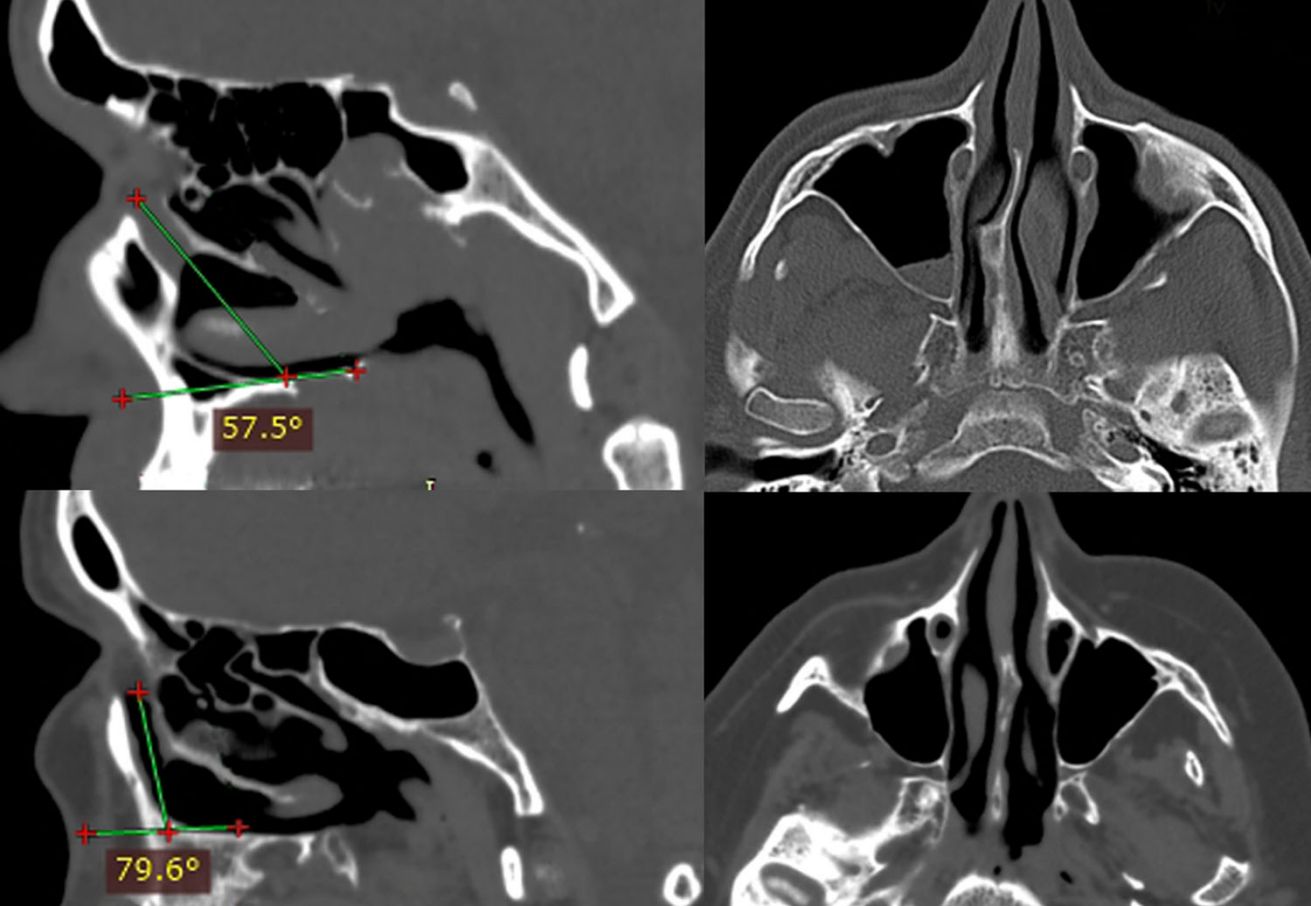
Slanted course of nasolacrimal duct (the angle between the duct and the palate 57.5°)—wide lacrimal recess (*top*). Vertical course of nasolacrimal duct (the angle between the duct and the palate 79.6°)—lacrimal recess is missing (*bottom*)

The distance from anterior maxillary wall to the nasolacrimal bony canal (the width of lacrimal recess) on the left and on the right side was measured, at the level of the inferior turbinate attachment to the lateral wall of the nasal cavity, by two of the authors using calipers on a computer screen (Fig. 1). The slope of the nasolacrimal canal was evaluated by measuring the angle between long axis of the canal and the horizontal plane of the hard palate (Fig. 2).

The proportion of cases with favorable anatomical conditions to perform minimally invasive middle maxillectomy was assessed. To determine feasibility of this procedure (opening the sinus endoscopically through lacrimal recess), it was presumed that the minimal necessary distance from anterior maxillary wall to nasolacrimal duct should be greater than 4 mm, just enough to create an ostium that could readily accept the 4 mm rigid endoscope.

Chi square test was used to determine the differences between men and women groups. The Spearman test was applied to assess the correlation between the slope of the nasolacrimal canal and the width of the lacrimal recess. A *P* value of less than 0.05 was considered statistically significant.

## Results

The mean distance between anterior maxillary wall and nasolacrimal bony canal as measured at the level of inferior turbinate attachment was 4.00 mm (range 0–15.2 mm) and was similar on both sides (left on average 4.69 mm, range 0–15.1 mm; right on average 4.92, range 0–15.2 mm). In males, the lacrimal recess width was significantly larger (on average 4.81 mm, range 0–15.2 mm) than in females (on average 3.40 mm, range 0–8.3 mm), *p* < 0.05, Chi square test.

**Fig. 3:**
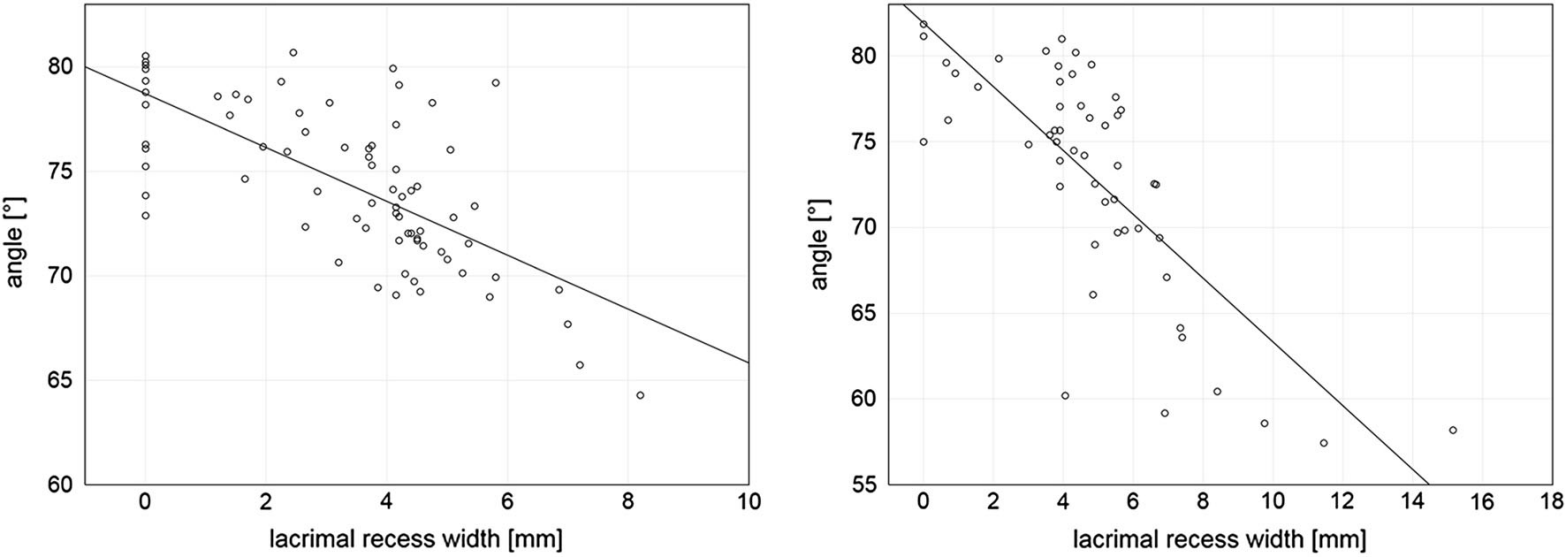
Scatterplot shows the slope of nasolacrimal canal (the mean values of the *left* and the *right side* angle between *long axis* of the canal and the *horizontal plane* of the hard palate) versus the width (mean values of the *left* and the *right side*) of lacrimal recess in females (*left*) and males (*right*). A negative correlation was found in both groups

The left and right nasolacrimal canal slope downwards, usually parallel to each other. In males, the angle between long axis of the canal and the horizontal plane of the hard palate varied between 57.5° and 81.6° (on average 73.3°) on the left side and 57.4°–82.1° (on average 72.4°) on the right side. In females, the angle ranged from 64.7° to 80.4° (on average 74.7°) on the left side and 63.9°–80.7° (on average 73.9°) on the right side. The Spearman test revealed negative correlation between the slope of the nasolacrimal canal and the width of the lacrimal recess in both sexes (*P* < 0.05). The more the vertical course of nasolacrimal duct, the narrower the lacrimal recess (Figs. 2, 3).

The proportion of cases with lacrimal recess wider than 4 mm was 69.6% (*n* = 87), and was higher in males 82.7% (*n* = 43) than in females 60.3% (*n* = 44). The difference between both groups was statistically significant (*P* < 0.05, test Chi Square).

In 30.4% of cases (*n* = 38), unfavorable anatomic conditions for minimally invasive medial maxillectomy were found—lacrimal recess was narrower than 4 mm. This situation was more frequent in females—39.7% of cases (*n* = 29) than in males 17.3% (*n* = 9). The difference between both groups was statistically significant (*P* < 0.05, Chi square test).

This group contained also 18 cases (14.4%), 6 men (11.5%) and 12 women (16.4%), in whom there was virtually no space between anterior maxillary wall and nasolacrimal canal; the measured distance between these two structures was 0 mm.

## Discussion

There is still a growing interest in minimizing surgical trauma accompanying endoscopic sinus surgery. As a result, several minimally invasive modifications of medial endoscopic maxillectomy have been proposed recently with the least traumatic techniques utilizing the lacrimal recess as a route to enter the sinus. Although there have been a number of reports published so far on the surgical anatomy of the nasolacrimal duct [7–9], none of them describes the anatomical variability of the position of nasolacrimal duct in relation to the anterior maxillary wall which is crucial for this type of approach.

Our study demonstrates that the minimally invasive medial maxillectomy approach leading through medial maxillary wall anteriorly to nasolacrimal duct is not always possible. In about 30.4% of cases, the distance between nasolacrimal canal and anterior maxillary wall (the width of lacrimal recess) is less than 4 mm, thus precluding creating a window that can accept a 4 mm endoscope and surgical instruments without damaging the piriform aperture rim or bony framework of nasolacrimal duct. The unfavorable anatomic conditions are more likely to occur in women. This may be attributable to the greater antero-posterior dimension of maxillary sinus in men as compared with that in women [10]. On the other hand, however, nasolacrimal duct slopes downward and posteriorly from lacrimal fossa to the inferior nasal meatus at a certain angle that varies between individuals. It was conspicuous in our patients that the more perpendicular the axis of the canal to the nasal flor the narrower the lacrimal recess. At the beginning of its course, in the vicinity of lacrimal fossa, nasolacrimal duct is always situated close to the anterior maxillary wall. Further downward, these two structures start to diverge making the lacrimal recess triangular in shape with the broadest diameter at the level of inferior turbinate. This is exactly the site where antrostomy is created during endoscopic minimally invasive medial maxilectomy.

Our study revealed that about 14.4% of individuals actually lack the lacrimal recess or there is hardly any space between the nasolacrimal duct and anterior wall of the maxillary sinus. In these patients, endoscopic minimally invasive approach (prelacrimal approach) cannot be performed. Presumably, these patients with more vertical nasolacrimal canal adherent to anterior maxillary wall are at greater risk of injury of nasolacrimal duct when anterior maxilectomy is performed either using endoscopic or Caldwell-Luc approach. On the other hand, whenever exploration of the anterior part of the sinus is necessary, owing to the anterior position of the nasolacrimal duct standard middle meatal antrostomy can be readily enlarged more in an anterior direction, thus facilitating visualization, surgical manipulation and removal of pathologic tissue from that region. In patients with slantwise course of nasolarimal canal, wide lacrimal recess can be expected and there is always more space that may remain out of reach through standard middle antrostomy. From the surgical point of view the wider the lacrimal recess, the more easily it can be explored using prelacrimal approach and the more reasonable this approach is in patients with pathology located in anterior part of maxillary sinus.

The medium sized lacrimal recess—less than 4 mm in width but still existing (measured distance 1–3 mm)—is the worst scenario for the surgeon. In this situation, because the distance between nasolacrimal bony canal and anterior wall of maxilla is too small to accept 4 mm optic, the total mobilization of nasolacrimal duct after removal of its bony framework may facilitate insertion the endoscope into the sinus; otherwise, anterior maxillectomy should be considered. Both solutions, however, increase intraoperative trauma.

## Conclusion

The course and position of nasolacrimal canal in relation to anterior maxillary wall vary between individuals, thus making the width of nasolacrimal recess ranging between 0 and 15.2 mm, as measured on the level of inferior turbinate attachment.

Careful analysis of individual anatomical conditions is recommended when minimally invasive medial maxillectomy is planned as in about 30% of cases the lacrimal recess is so narrow that this type of approach might be difficult to perform without damaging the piriform aperture rim or bony framework of nasolacrimal duct. In some patients, the lacrimal recess is missing making this type of approach impracticable.
